# Pharmacogenomic variabilities in geo-ancestral subpopulations and their clinical implications: Results of collaborations with Hmong in the United States

**DOI:** 10.3389/fgene.2022.1070236

**Published:** 2023-01-04

**Authors:** Boguang Sun, Ya-Feng Wen, Kathleen A. Culhane-Pera, Muaj Lo, Robert J. Straka

**Affiliations:** ^1^ Department of Experimental and Clinical Pharmacology, College of Pharmacy, University of Minnesota, Minneapolis, MN, United States; ^2^ Minnesota Community Care, St. Paul, MN, United States

**Keywords:** pharmacogenomics, minority health, health disparities, community-based participatory research, Hmong, Asian American, personalized medicine

## Abstract

Underrepresentation of subpopulations within geo-ancestral groups engaged in research can exacerbate health disparities and impair progress toward personalized medicine. This is particularly important when implementing pharmacogenomics which uses genomic-based sources of variability to guide medication selection and dosing. This mini-review focuses on pharmacogenomic findings with Hmong in the United States and their potential clinical implications. By actively engaging Hmong community in pharmacogenomic-based research, several clinically relevant differences in allele frequencies were observed within key pharmacogenes such as *CYP2C9* and *CYP2C19* in Hmong compared to those in either East Asians or Europeans. Additionally, using state-of-the-art genome sequencing approaches, Hmong appear to possess novel genetic variants within *CYP2D6*, a critical pharmacogene affecting pharmacokinetics of a broad range of medications. The allele frequency differences and novel alleles in Hmong have translational impact and real-world clinical consequences. For example, Hmong patients exhibited a lower warfarin stable dose requirement compared to East Asian patients. This was predicted based on Hmong’s unique genetic and non-genetic factors and confirmed using real-world data from clinical practice settings. By presenting evidence of the genetic uniqueness and its translational impact within subpopulations, such as the Hmong, we hope to inspire greater inclusion of other geo-ancestrally underrepresented subpopulations in pharmacogenomic-based research.

## 1 Introduction

Individual responses to medications can vary, in part due to genomic variability (Roden et al., 2011). Pharmacogenomics (PGx) focuses on the use of individuals’ genomic information to guide medication selection and dosing in order to maximize therapeutic benefits and minimize side effects (Sadee and Dai, 2005). Over the past few decades, incorporation and application of PGx information into clinical decisions have been on the rise. So far, the Clinical Pharmacogenetics Implementation Consortium (CPIC) has published 26 clinical guidelines to help clinicians systematically interpret genetic tests to optimize medication therapy (CPIC, 2021). Also, around 15% of the medications approved by the US Food and Drug Administration (FDA) contain PGx-guided recommendations on their respective package inserts (FDA, 2022).

Individuals’ genomic information can differ significantly based on their geo-ancestral origins (Slatkin and Racimo, 2016). In recent years, in response to the US National Institutes of Health “All of Us” initiative (All of Us Research Program et al., 2019), the importance of including diverse populations in PGx studies has been gaining recognition (Mensah et al., 2019; Magavern et al., 2022). A 2009 study concluded that 96% of participants in genome-wide association studies (GWAS) were of European ancestry (Need and Goldstein, 2009). A subsequent 2016 study showed that 80% participants in GWAS were of European ancestry, due to more studies being conducted in East Asian countries such as China and Japan (Popejoy and Fullerton, 2016). As a broad geographical area, East Asia consists of subpopulations with diverse cultural and genomic backgrounds, such that differences in allele frequencies in single nucleotide variations (SNVs) exist among East Asian subpopulations (Lo et al., 2020; Pan and Xu, 2020).

In the US, there is still a paucity of PGx research being conducted in minority East Asian subpopulations. Examples of minority East Asian subpopulations in the US include Hmong, Filipinos, Cambodians, and Vietnamese (United States Census Bureau, 2019). If clinical decisions incorporating PGx are made based on the patient’s general East Asian ethnic category, the potential risks of failing to appreciate unique allele variations within minority Asian subpopulations could result in inappropriate medication recommendations. This underscores the critical need to expand inclusion of diverse populations in PGx research, specifically focusing on underrepresented subpopulations.

In this article, we highlight our accumulated research endeavors that collaborated with Hmong community to identify unique allele frequencies and novel SNV’s within key pharmacogenes. Translating such observations into real-world clinical decision-making tools holds promise for more safe and effective use of medications to achieve improved clinical outcomes.

## 2 Hmong in the US

The Hmong are an East Asian subpopulation that originated in China and migrated into Southeast Asia in the 1800s. After the end of the Vietnam War, over 150,000 people fled Laos for Thailand, where they were re-settled as refugees in multiple countries (Goldfarb, 1982). Now the Hmong diaspora includes the United States, France, Australia, French New Guinea, as well as China, Laos, Thailand, and Vietnam. The most populous states in the US are California, Minnesota, and Wisconsin. In 2019, the population of Hmong in the United States was 326,843 and Minnesota had 94,079 Hmong (United States Census Bureau, 2019).

The Hmong are experiencing a high prevalence of chronic diseases including gout, hypertension and type 2 diabetes, and in some cases higher rates compared to other populations (Culhane-Pera et al., 2009; Wahedduddin et al., 2010; Thao et al., 2015; Lor, 2018; Roman et al., 2021). Research has been conducted to investigate factors contributing to this observation as well as the overall quality of medical care for chronic diseases. Possible factors include general healthcare screening seeking behavior (Barrett et al., 1998), health literacy (Wong et al., 2005), traditional beliefs (Helsel et al., 2005), cultural clashes with the health care system and concerns about taking chronic medications (Culhane-Pera et al., 2003), and environment and lifestyle changes. A recent cross-sectional study explored how migration from Southeast Asia to US with the changes in environment and lifestyle, including diet, could significantly decrease the diversity and function of Hmong gut microbiota, which in turn could contribute to obesity and cardiometabolic disease progression (Vangay et al., 2018). In addition, recent discoveries of relatively unique prevalence of SNVs known to affect medication selection and dosing may be contributing, which represent the prime focus of contemporary PGx research.

## 3 Genomics and PGx research in Hmong

Hmong appear to be a genetically unique population, which is supported by multiple studies. Hmong can be differentiated from Thai and Chinese based on only 32 microsatellite genetic markers in contrast with the absence of a clear separation between Thai and Chinese individuals (Listman et al., 2007). Hmong are genetically and linguistically the most distinct ethnic group compared to four other Thai hill tribe populations (Akha, Karen, Lahu, and Lisu), as indicated by a study analyzing 2,445 genome-wide single nucleotide polymorphism markers in the Human Genome Diversity Project (Listman et al., 2011). Additionally, excess rare alleles have been found in the Hmong population that could be explained by accumulated mutations over time or through genetic drift caused by significant losses in the Hmong population due to wars (Listman et al., 2011). Analysis of 537 Hmong-Mien speaking individuals using mitochondrial DNA demonstrated variable ratios of lineages that implied Hmong-Mien populations had more genetic contacts with Northern East Asians compared to Southern East Asians (Wen et al., 2005; Yang et al., 2022). Hmong kinship practices could contribute as well, including clan hospitality (treating people with the same last name as family), clan exogamy (marrying outside one’s clan), ethnic endogamy (marrying within one’s ethnic group) (Lee, 2020), as well as cross-cousin marriage (Culhane-Pera et al., 2003). All of these observations provide a basis for conducting genomic research in this genetically unique population.

Using principles of community-based participatory research (Culhane-Pera et al., 2017b), our research efforts have engaged Hmong to participate in PGx studies in the past 15 years. After exploring cultural concepts of heredity and reactions to genetic research (Culhane-Pera et al., 2017a), PGx studies were conducted in two independent cohorts of Hmong adults (age ≥ 18 years old) in Minnesota. The first cohort enrolled 236 self-identified Hmong (*Hmong-PGx cohort*) in 2009 (Straka et al., 2010). The second cohort enrolled 198 Hmong (*VIP-Hmong cohort*) in 2017 (Wen et al., 2020). Collectively, a selection of SNVs within key pharmacogenes were identified to exhibit differential frequencies between Hmong and East Asians, including CYP2C9*3 (18.9% vs. 3%), CYP2C19*2 (42.2% vs. 29%), CYP2C19*3 (0.3% vs. 8.3%), and CYP4F2*3 (6.5% vs. 22.1%) (Wen et al., 2020; Sun et al., 2021). Such differences could have translational impact on medication selection and dosing for Hmong. Based on the CPIC guidelines (CPIC, 2021), compared to other East Asians, Hmong would require alternative therapies for several medications based on their predicted phenotypes. Examples of medications include antithrombotic agents such as warfarin and clopidogrel, and antidepressants such as selective serotonin reuptake inhibitors (SSRIs) and tricyclic antidepressants (TCAs) (Wen et al., 2020).

Other than the apparent differences in allele frequencies of SNVs, unique subpopulations also have novel genetic variants, which appears to be the case for the Hmong related to genetic variants within a key pharmacokinetics (Woodahl et al., 2014) gene: *CYP2D6*. This highly polymorphic pharmacogene has an impact on several medications including tamoxifen, SSRIs and opioids (CPIC, 2021). Using targeted next-generation sequencing with confirmatory Sanger sequencing, three novel sub-alleles (*10.007, *36.004, and *75.002) were identified (Wen et al., 2022b). This discovery underscores the importance of using novel sequencing techniques to better characterize genomic profiles in subpopulations in addition to reporting allele frequencies in common alleles.

## 4 Clinical translations and impact

By using available tools such as CPIC guidelines, FDA package inserts or other published PGx-guided dosing algorithms, research has been conducted to further demonstrate the clinical translation and implications of these unique allele frequency distributions discovered in our Hmong cohorts. The following sections describe examples applied to several classes of medications.

### 4.1 Anticoagulants and antiplatelet agents

Warfarin is a commonly used anticoagulant that exhibits high degree of inter-individual dosing variability (Johnson et al., 2017). Validated factors determining warfarin dosing include clinical factors such as medical/medication histories, demographic factors such as patients’ age, weight and height, and genetic factors such as SNVs in *CYP2C9*, *VKORC1* and *CYP4F2* (Gage et al., 2017). Based on FDA PGx-guided warfarin dosing recommendation that incorporates *CYP2C9* and *VKORC1* genotypes, a higher percentage of Hmong (N = 433) was estimated to be very sensitive (require only 3.5–14 mg/week warfarin dose) to warfarin compared to East Asians (28% vs. 5%) (Coumadin, 2021; Sun et al., 2021). Furthermore, using the International Warfarin Pharmacogenetics Consortium algorithm that considers both genetic and non-genetic factors in warfarin dosing, Hmong (N = 433) were predicted to require a significantly lower mean warfarin stable dose compared to East Asians (19.8 vs. 21.3 mg/week) (International Warfarin Pharmacogenetics Consortium et al., 2009; Sun et al., 2021).

In addition to prediction of translational significance, real-world evidence was investigated to support the predicted warfarin dosing difference between Hmong and East Asian patients. Using Minnesota Fairview Hospitals’ electronic health records of 55 Hmong and 100 East Asian long-term warfarin users, the mean warfarin stable dose was significantly lower in Hmong than in East Asian patients (14.5 vs. 20.4 mg/week) (Sun et al., 2022). Furthermore, compared to East Asian patients, Hmong patients had 3.1-fold higher hazard of the composite outcome including International Normalized Ratio>4 and major bleeding events (Sun et al., 2022). These observations corroborate the previous prediction on warfarin stable dose based on Hmong’s genetic and non-genetic factors, and further underscore the need to develop a Hmong specific warfarin PGx dosing algorithm.

Another clinically relevant issue pertains to *CYP2C19* phenotypes and selection of clopidogrel over other P2Y12 inhibitor antiplatelet agents such as ticagrelor and prasugrel (Lee et al., 2022). Clopidogrel’s activation and therapeutic effects are highly dependent on an individual’s *CYP2C19* phenotype (Plavix, 2022). Individuals determined to be poor metabolizers of *CYP2C19* should consider alternative agents other than clopidogrel (FDA Boxed Warning) (Plavix, 2022). East Asians in general have a higher prevalence of *CYP2C19* poor metabolizers compared to Europeans (13% vs. 2.4%) (Lee et al., 2022). Combining our *Hmong-PGX* and *VIP-Hmong* cohorts (N = 389), Hmong have a significantly higher prevalence of *CYP2C19* poor metabolizers compared to East Asians (17.5% vs. 13%). As such, the Hmong are more likely to experience supratherapeutic treatment outcomes from clopidogrel.

### 4.2 Gout and allopurinol uses

Allopurinol is the most commonly used urate-lowering therapy, but it exhibits large inter-individual variability in its PK and Pharmacodynamics (PD) (Rundles, 1969). The *SLC22A12* gene, encoding URAT1, contributes to high variability in plasma oxypurinol, the key active metabolite of allopurinol. URAT1 plays an important role in urate and oxypurinol reabsorption at the proximal renal tubule (Emmerson et al., 1987; Iwanaga et al., 2005). In contrast to *SLC22A12*, the *ABCG2* gene, encoding BRCP, is a key transporter modulating the serum urate (SU)-lowering response to allopurinol (Wen et al., 2015; Roberts et al., 2017; Wallace et al., 2018; Brackman et al., 2019). Other genes may impact the PK and PD of allopurinol but with limited evidence, including *AOX1*, *MOCOS*, and *GREM2* (Roberts et al., 2010; Brackman et al., 2019). In an open-labeled PGx-guided study of allopurinol in Hmong adults with gout or hyperuricemia (N = 34), *SLC22A12* rs505801C>T was identified to be associated with the PK of oxypurinol (Roman et al., 2017). However, *ABCG2 rs2231142C>A* was not associated with the PD of oxypurinol (Roman et al., 2017). When applying non-linear mixed effect analysis, genetic variants in *SLC22A12* were identified to be associated with oxypurinol clearance and variants in *PDZK1* were associated with urate-lowering effect of oxypurinol (Wen et al., 2022a). Based on these results, the allopurinol maintenance dose needed to achieve target SU level in Hmong can be predicted using an individual’s body mass, renal function, and now, possibly genetic variants in *SLC22A12* and *PDZK1* (Wen et al., 2022a).

HLA-B*58:01 has demonstrated a strong predictive value for allopurinol-associated severe cutaneous adverse reactions (Lerch et al., 2018), particularly for Koreans, Han Chinese or Thai (FitzGerald et al., 2020). Given a recognized higher incidence and severity of gout in Hmong vs. other populations (Wahedduddin et al., 2010), HLA-B*58:01 carrier status in Hmong (N = 95) was quantified. The results showed that the percentage of HLA-B*58:01 carriers in Hmong was significantly lower than Han Chinese and Koreans (2.1% vs. 19.6% vs. 12.2%) (Peng et al., 2020). Although preliminary, these findings suggest the recommendation of HLA genotyping for East Asian populations (FitzGerald et al., 2020) may warrant reconsideration for East Asian subpopulations.

### 4.3 Antidepressants

Mental health disorders are often untreated and unrecognized (Sin et al., 2011), perhaps even more so in the Hmong community (Collier et al., 2012). The current practice of trial-and-error methods to discover effective antidepressants may further exacerbate the disparities. In general, only 37% of patients achieve remission by 8 weeks after the first treatment (Sinyor et al., 2010; APA, 2022). Many anti-depressant medications, including SSRIs and TCAs, primarily undergo biotransformation by *CYP2C19* and/or *CYP2D6*. PGx-guided treatment for depression has demonstrated improved remission rates versus usual-care in European populations (Rosenblat et al., 2018). When investigating how PGx may impact antidepressant treatment recommendations in Hmong participants (N = 12) using a comprehensive PGx panel (RightMedTM, OneOme), it was found that moderate or major “gene-drug interactions” between *CYP2C19* and/or *CYP2D6* with at least one antidepressant was predicted in all participants. This observation resulted in “choose alternate medication” recommendation for 11/16 investigated antidepressants (Nelson et al., 2020). If individualized PGx is applied more broadly in clinical practice, there may be opportunities for improved selection and use of antidepressants that translate into better outcomes for subpopulations exhibiting unique profiles of SNVs.

## 5 Other examples supporting the inclusion of geo-ancestrally diverse population

Failure to recognize the unique geo-ancestral differences in PGx can have real-world ramifications. The overall minor allele frequency (MAF) of a specific SNV can modulate the translational impact of SNVs on medication selection and dosing. A well-known example relates to the CYP2C9*5, *6, *8, and *11 alleles in warfarin dosing, which are prevalent among African populations and virtually non-existent in non-African populations (Perera et al., 2014). Failure to incorporate these alleles in warfarin PGx dosing algorithms led to inferior performance of such algorithms in patients of African ancestry (Kimmel et al., 2013).

As another example, *CYP2C19* poor/intermediate metabolizers of clopidogrel vary substantially among different populations ranging from 58% in East Asians to 28% in Europeans (Lee et al., 2022). This can partially explain the lack of statistical power to ascertain the advantage of implementing PGx-guided approach for P2Y12 inhibitors selection compared to the current clinical practice in large-scale landmark clinical trials that primarily consisted of Europeans such as the POPular Genetics trial (Claassens et al., 2019). Consequently, the thresholds of the MAF for clinical actionable SNVs, which are set primarily based on patients of European ancestry, may mask the real-world frequency of such SNVs in diverse populations and hence impede, or at least, confound the translation and implementation of PGx in clinical settings. Thus, there is a critical need to assess the real-world applicability and clinical usability of current PGx guidelines in geo-ancestrally diverse populations.

## 6 Discussion and future directions

Hmong, along with other underrepresented subpopulations (Fohner et al., 2013; Woodahl et al., 2014; Leitch et al., 2022), are eager for clinicians to recognize their uniqueness when considering selection and dosing of medications, rather than being represented by the broader definition of geo-ancestral grouping (Culhane-Pera et al., 2017a; Culhane-Pera et al., 2017b; Holzer et al., 2021). PGx has uncovered some relevant issues, but more PGx research is needed within subpopulations to identify differences in genomic biomarkers that can contribute to personalized medicine. It should be acknowledged that conducting research within underrepresented communities can have particular challenges. We encourage researchers to 1) actively engage with liaisons and leaders from the community to discuss key community needs and how research can assist in addressing such needs; 2) create community-academic research teams to ensure that methods are appropriate and effective for the community; and 3) disseminate the findings back to the community in culturally and linguistically appropriate ways. These approaches can contribute to maximizing benefits that communities can gain from participating in PGx research.

### 6.1 Future direction 1: Expand PGx research with Hmong community in the US

Discovery of unique allele frequencies and novel SNV’s in Hmong from the Midwest should ideally be validated with Hmong from other regions of the US. This would provide relative assurance that our observations are more widespread and relevant to those who identify as Hmong within the US. To that end, ongoing PGx studies in the Central Valley of California [where the largest Hmong population resides in the US (Lor, 2018)] will increase PGx knowledge and clinical understanding. At this stage, two recruitment events were successfully completed by our group in Fresno, and Sacramento, California with analysis forthcoming.

### 6.2 Future direction 2: Explore other factors affecting Hmong’s health and medication uses

PGx research results were described as “a positive light” for the community by Hmong research participants (Holzer et al., 2021). Indeed, however caution regarding the use of genomic information alone to guide drug therapy was advised, and consideration of non-genetic factors when interpreting PGx was stressed. The role of diet and gut microbiome were specifically explored, as Hmong “eat differently” from other non-Hmong, which they perceive could complicate their medication responses and disease progression. Recently, a Phase-II clinical trial was conducted by our group aiming to decipher the relationship between gout incidence and microbiome, obesity, genomic and clinical risk factors in Hmong (National Library of Medicine., 2021). This integrative approach incorporating PGx and other multi-omics biomarkers may elucidate causes of severe gout in Hmong men.

## 7 Conclusion

This mini-review highlights the significant and translational impact of variability in PGx in Hmong population, as a geo-ancestral subpopulation, discovered by our collaboration with Hmong community. The current challenges and future opportunities were presented with the goals of expanding diversity in PGx research and reducing health disparities. Finally, it is expected that our approaches to conducting and communicating PGx research in Hmong community can be motivating for other researchers.
